# Notoginsenoside R1, a Metabolite from *Panax notoginseng*, Prevents Paclitaxel-Induced Peripheral Neuropathic Pain in Mice

**DOI:** 10.3390/molecules30173613

**Published:** 2025-09-04

**Authors:** Muneerah Al-Musailem, Willias Masocha, Altaf Al-Romaiyan

**Affiliations:** 1 Master of Science in Pharmaceutical Sciences Program, College of Pharmacy, Kuwait University, Safat 13110, Kuwait; muneerah.almusailem@ku.edu.kw; 2 Department of Pharmacology & Therapeutics, College of Pharmacy, Kuwait University, Safat 13110, Kuwait; willias.masocha@ku.edu.kw

**Keywords:** paclitaxel, peripheral neuropathy, plant extract, triterpenoid, mechanical allodynia, monoacylglycerol lipase activity

## Abstract

Development of paclitaxel-induced neuropathic pain (PINP) during chemotherapy may lead to paclitaxel discontinuation, potentially compromising effective anticancer therapy. PINP can manifest as allodynia. One recently discovered key factor in paclitaxel-induced mechanical allodynia (PIMA) pathogenesis is the elevated activity of monoacylglycerol lipase (MAGL), an enzyme that metabolizes the endocannabinoid 2-arachidonoylglycerol (2-AG). Thus, inhibiting MAGL serves as a potential analgesic target. Notoginsenoside R1 (NGR1), a metabolite of *Panax notoginseng*, has shown promise in reducing oxidative stress and neuronal apoptosis in nerve injury models. However, its effects on PIMA and MAGL activity have not yet been explored. This study is a proof-of-concept preclinical study investigating the antiallodynic effects of NGR1 on PIMA in female BALB/c mice and also examining its effect on MAGL activity. The effect of treatment of mice with NGR1 intraperitoneally on the development of PIMA was evaluated. Molecular docking using CB-Dock2 compared the binding energies to MAGL of NGR1 and pristimerin, a triterpene MAGL inhibitor. The effects of NGR1 on human recombinant MAGL activity, as well as the MAGL activity in mice paw skin tissues, were assessed using MAGL inhibitor screening and MAGL activity assay kits, respectively. NGR1 prevented the development of PIMA in a dose-dependent manner. The docking scores showed that NGR1 has a good affinity for MAGL (−7.8 kcal/mol, binding energy) but less affinity than pristimerin (−10.3 kcal/mol). NGR1 inhibited the human recombinant MAGL activity in a reversible and concentration-dependent manner, although the inhibition was in a reverse order. Treatment of mice with NGR1 showed a non-significant trend in reducing the paclitaxel-induced increase in MAGL activity in the paw skin. This study shows for the first time that NGR1 prevents the development of PIMA and suggests that NGR1 has affinity for and inhibits human recombinant MAGL activity with a paradoxical inhibition pattern. More mechanistic studies are needed to fully elucidate the molecular mechanisms of NGR1 in preventing PIMA.

## 1. Introduction

Chemotherapy-induced neuropathic pain (CINP) is a dose-limiting side effect of many chemotherapeutic agents including paclitaxel, a taxane effective in the treatment of many types of solid tumors such as breast cancer, non-small cell lung cancer, and ovarian cancer. CINP symptoms, such as thermal hyperalgesia, cold and mechanical allodynia, and spontaneous sensations, such as burning, prickling, shooting, spasm, and numbness, can be debilitating, often necessitating dose adjustments or treatment discontinuation, all of which can compromise optimal cancer management [1]. This could significantly diminish patients’ quality of life [2]. Despite numerous efforts to prevent and treat CINP through medications, only duloxetine shows moderate efficacy in the management of established CINP. Duloxetine is a selective serotonin and norepinephrine reuptake inhibitor originally used to treat depression and now used for treating neuropathic pain. The lack of an adequate response to duloxetine in most patients has highlighted the need to investigate new effective preventative or treatment agents for the management of CINP [3].

The pathogenesis of CINP involves multiple mechanisms including the dysregulation of endocannabinoid system (ECS). The ECS is a widely acknowledged neuromodulator network whose dysregulation has been shown to contribute to CINP [4]. It comprises cannabinoid receptors (primarily CB1 and CB2), endogenous ligands such as 2-arachidonoyl-glycerol (2-AG) and N-arachidonoylethanolamine (AEA), and the enzymes responsible for 2-AG and AEA degradation such as monoacylglycerol lipase (MAGL) and fatty-acid amide hydrolase (FAAH), respectively. 2-AG exerts its effects through CB1 and CB2 activation. Its signaling is tightly regulated by catabolic enzymes, with MAGL serving as the major enzyme responsible for 2-AG degradation [5].

In CINP, several studies have shown a significant deficiency in peripheral endocannabinoids [6,7,8]. Paclitaxel treatment leads to a deficiency of 2-AG in the periphery, contributing to the development of paclitaxel-induced mechanical allodynia (PIMA) [8]. The decrease in 2-AG was largely attributed to the increased activity of MAGL in the paw skin of mice [9]. This led to the hypothesis that elevating local 2-AG levels, either through endocannabinoid administration or inhibition of MAGL, may relieve CINP. Indeed, restoring 2-AG levels via MAGL inhibition by JZL184, MAGL and FAAH inhibitor, has been shown to alleviate PIMA in a CB1/CB2 receptor-dependent manner [8]. We have also recently reported that pristimerin, a quinone methide triterpene plant extract, inhibits MAGL activity, increases the expression of *Nrf2*, a major antioxidant marker, and alleviates PIMA in female BALB/c mice [9].

**Figure 1:**
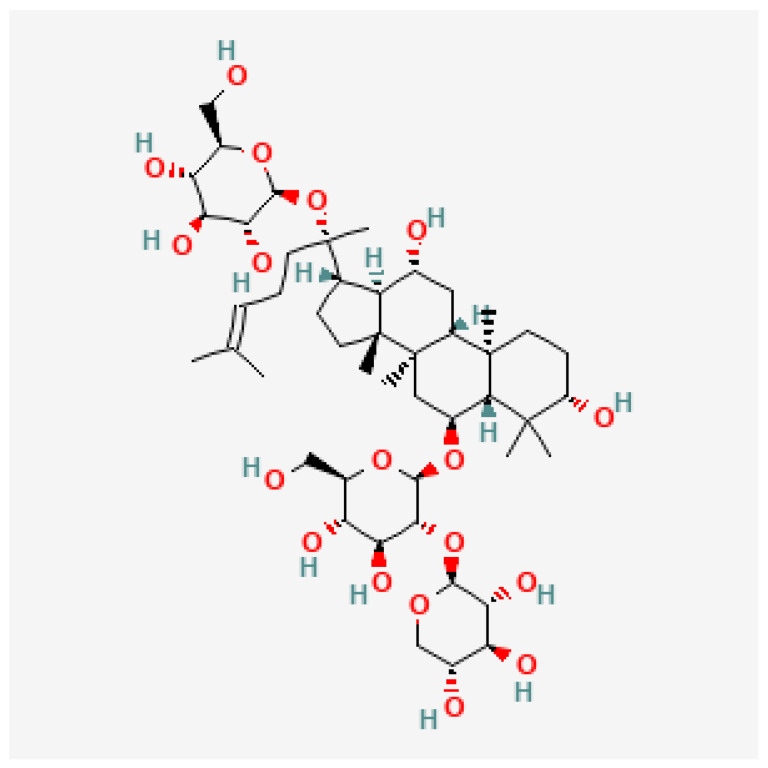
Chemical structure of Notoginsenoside R1. Extracted from [13].

Plant-derived extracts have attracted significant interest as potential adjunct or alternative therapeutic options for managing neuropathic pain including CINP [10,11]. Among these, notoginsenoside R1 (NGR1), a tetracyclic triterpenoid metabolite derived from *Panax notoginseng*, has gained attention for its neuroprotective abilities. The structural formula of notoginsenoside R1 is depicted in Figure 1. Studies have shown that NGR1 can mitigate oxidative stress, neuronal apoptosis, and inflammation in a rat spinal cord injury model [12]. However, despite its reported neuroprotective properties, the effects of NGR1 on CINP have not yet been evaluated in any animal model. Taking into consideration that NGR1 is a triterpene similar to pristimerin, we hypothesized that NGR1 could work in a similar fashion to pristimerin to inhibit PIMA. Therefore, the aim of this study was to investigate the antiallodynic potential of NGR1 in PIMA, a model of CINP, and to evaluate the effect of NGR1 on MAGL activity.

## 2. Results

### 2.1. Effect of NGR1 Pretreatment on Paclitaxel-Induced Mechanical Allodynia

**Figure 2:**
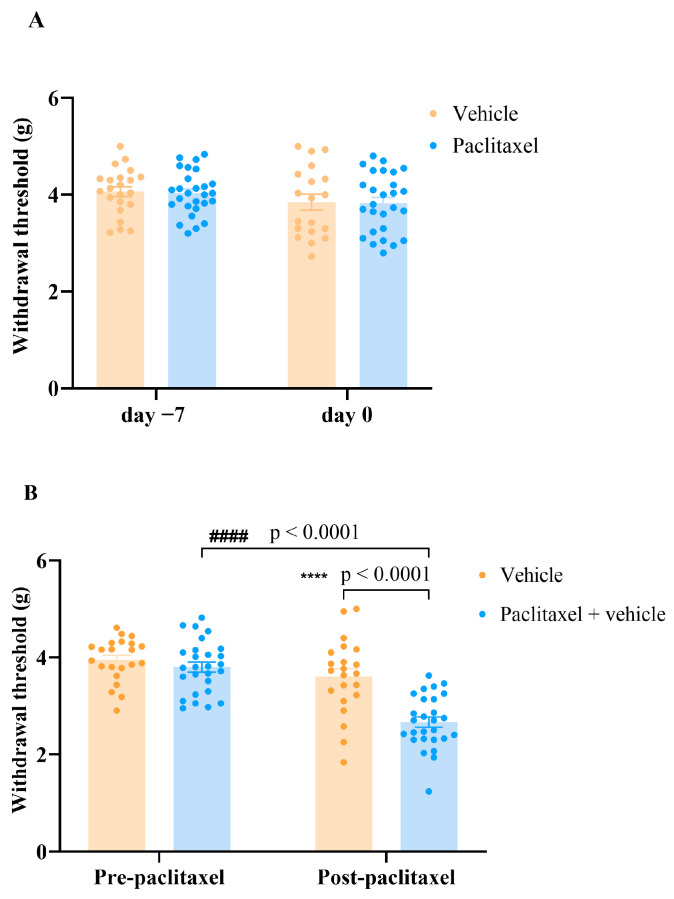
The development of paclitaxel-induced mechanical allodynia at day 7 after first injection of paclitaxel. (**A**) Withdrawal threshold to mechanical stimulus were measured in vehicle- and paclitaxel-treated female BALB/c mice at day −7 and day 0 before first paclitaxel injection. (**B**) Withdrawal thresholds to mechanical stimulus were measured pre-paclitaxel and post-paclitaxel (day 7 after first paclitaxel injection) in vehicle and paclitaxel groups. Each bar represents the mean ± S.E.M of values obtained from 22–27 animals per treatment group. **** *p* < 0.0001 as compared to paclitaxel group post-paclitaxel at the same time point and #### *p* < 0.0001 compared to paclitaxel group at baseline (pre-paclitaxel) (two-way ANOVA followed by Tuckey’s multiple comparison posttests).

The withdrawal threshold to mechanical stimuli was assessed for both the vehicle- and paclitaxel-treated groups on day −7 and day 0 before paclitaxel treatment (Figure 2A). On day −7, withdrawal thresholds were comparable between the vehicle group (4.06 ± 0.10 g) and the paclitaxel group (4.03 ± 0.09 g; *p* = 0.8235). This similarity persisted on day 0, prior to the first paclitaxel injection, with thresholds of 3.85 ± 0.17 g for the vehicle group and 3.83 ± 0.19 g for the paclitaxel group (*p* = 0.9156). Therefore, the data from day −7 and day 0 were combined to establish the pre-paclitaxel baseline (Figure 2B).

The pre-paclitaxel withdrawal thresholds were 3.95 ± 0.10 g in the vehicle group and 3.80 ± 0.11 g in the paclitaxel group. Following five consecutive days of paclitaxel administration, the post-paclitaxel withdrawal threshold in the paclitaxel group was significantly reduced to 2.67 ± 0.11 g, compared to the vehicle-treated group (3.61 ± 0.17 g; *p* < 0.0001) at the same time point (on the 7th day after first paclitaxel or its vehicle injection). Furthermore, within the paclitaxel group, a significant decrease in withdrawal threshold was observed from pre-paclitaxel (3.80 ± 0.11 g) to post-paclitaxel (2.67 ± 0.11 g; *p* < 0.0001; Figure 2B). Thus, paclitaxel treated mice developed PIMA.

**Figure 3:**
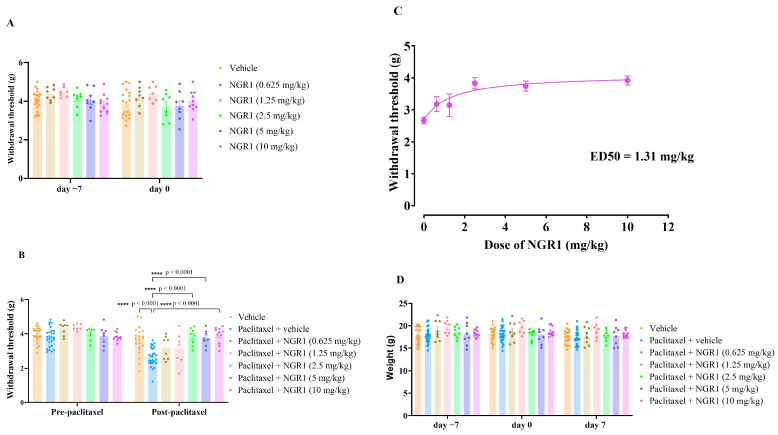
Prophylactic treatment of female BALB/c mice with NGR1 prevents the development of paclitaxel-induced mechanical allodynia at day 7 after the first injection of paclitaxel. (**A**) Withdrawal threshold to mechanical stimulus in paclitaxel plus NGR1 mice as compared to vehicle-treated counterparts at day −7 (before the first NGR1 injection) and day 0 (after five consecutive days of NGR1 injection) before the first paclitaxel injection. No significant changes in force intensity were observed for any of the groups. Data are presented as mean ± S.E.M, with *n* = 7–22 per experimental group. *p* > 0.05 as compared to the vehicle group (two-way ANOVA followed by Tukey’s multiple comparison test). (**B**) Mice were treated with different doses of NGR1 (0.625–10 mg/kg) twice daily for 5 days before and 5 days during paclitaxel administration. Withdrawal thresholds to mechanical stimulus were measured before and after paclitaxel administration. Each bar represents the mean ± S.E.M of values obtained from 7–27 animals per treatment group. **** *p* < 0.0001 as compared to the paclitaxel group post-paclitaxel (two-way ANOVA followed by Tukey’s multiple comparison test). (**C**) Dose–response curve of withdrawal threshold at day 7 after five days administration of paclitaxel following NGR1 administration (0.625–10 mg/kg twice daily for 5 days before and 5 days during paclitaxel administration). The curve indicates that increasing the dose of NGR1 resulted in an improvement in withdrawal threshold. Each point represents mean ± S.E.M of values obtained from 7–27 animals per treatment group, (curve fit, [agonist] vs. response [3 parameters]). (**D**) Body weight (g) measurement at day −7, day 0 and day 7 of vehicle, paclitaxel, and paclitaxel + NGR1 (doses ranging from 0.625–10 mg/kg) groups. No significant differences in weight were observed for each group during the entire experiment. Data are mean ± S.E.M of values obtained from 7–27 animals per treatment group. *p* > 0.05 as compared to the vehicle group at the same time point (two-way ANOVA followed by Tukey’s multiple comparison test).

Administration of NGR1 (0.625–10 mg/kg i.p.) twice daily before the start of paclitaxel treatment did not result in any significant change in the withdrawal threshold to mechanical stimuli on day −7 (before the first NGR1 treatment) compared to day 0 (after five consecutive days of NGR1 injections), nor when compared to the vehicle group (Figure 3A). This suggests that NGR1 alone (up to 10 mg/kg administered twice daily) had no effect on the withdrawal threshold of mice as compared to the vehicle group before paclitaxel administration, indicating that NGR1 alone did not have an effect on normal mice response to mechanical stimuli.

The administration of NGR1 twice daily for five consecutive days before the first administration of paclitaxel and concomitantly with paclitaxel injection for another five consecutive days significantly prevented the development of PIMA at day 7 after the first paclitaxel injection in female BALB/c mice (Figure 3B). Co-treatment with NGR1 prevented the paclitaxel-induced reduction in the mechanical withdrawal threshold of mice in a dose-dependent manner. Mice treated with paclitaxel plus NGR1 had withdrawal thresholds similar to mice treated with the vehicles only (paclitaxel plus NGR1 2.5 mg/kg: 3.83 ± 0.18 g; paclitaxel plus NGR1 5 mg/kg: 3.74 ± 0.16 g; and paclitaxel plus NGR1 10 mg/kg: 3.92 ± 0.14 g vs. vehicles only: 3.60 ± 0.17 g; *p* > 0.05). Furthermore, mice treated with paclitaxel plus NGR1 had withdrawal thresholds that were significantly higher than those treated with paclitaxel plus the vehicle (paclitaxel plus vehicle: 2.67 ± 0.11 g; *p* < 0.0001 as compared to the paclitaxel group post-paclitaxel). The dose–response curve showed that NGR1 improved the withdrawal threshold of paclitaxel-treated animals in a dose-dependent manner (Figure 3C), with an ED_50_ of 1.31 mg/kg. Treating mice with the vehicle, paclitaxel and NGR1 was not associated with reduction in body weight at all time points assessed (Figure 3D).

### 2.2. Affinity of NGR1 to MAGL

**Figure 4:**
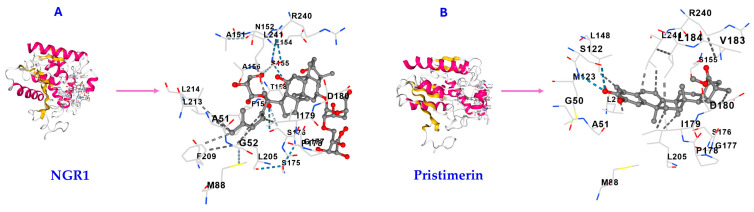
Molecular docking results of NGR1 and pristimerin on MAGL. The interactive visualization for the 3D structure of (**A**) MAGL with NGR1 bound to the cavity and (**B**) MAGL with pristimerin bound to the cavity. Both NGR1 (**A**) and pristimerin (**B**) bind MAGL on the same amino acids and binding pocket. Images obtained from molecular docking performed using the CB-Dock2 server.

The molecular docking results, based on the Vina scores, show higher binding energy (lower affinity) of NGR1 to MAGL (−7.8 kcal/mol) compared to the triterpene pristimerin (−10.3 kcal/mol, Table 1), which is a known MAGL inhibitor [9,14]. NGR1 interacted with all the amino acids on MAGL that pristimerin interacted with on MAGL, although it interacted with extra amino acids, which pristimerin did not interact with (Table 1). This suggests NGR1 has good affinity for MAGL on the same binding pocket (Figure 4) as pristimerin, although with lower affinity.

### 2.3. Inhibitory Effects of NGR1 on Recombinant Human MAGL Activity

**Figure 5:**
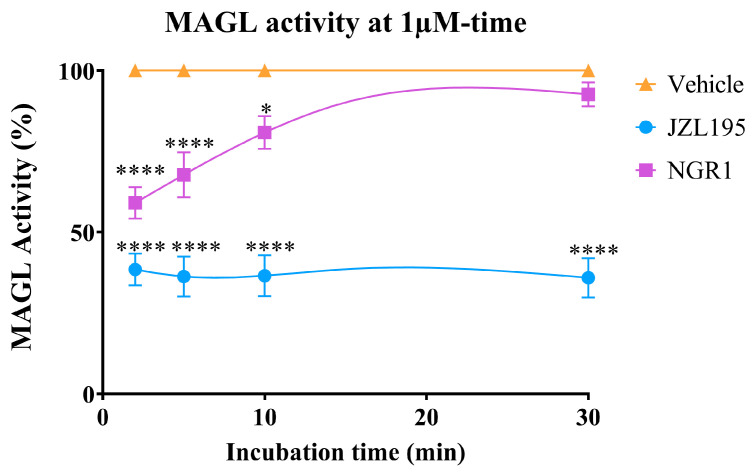
Inhibitory effects of 1 μM NGR1 and JZL195 on human recombinant MAGL activity over time. NGR1, at a concentration of 1 μM, shows a reversible MAGL inhibitory effect over time in comparison to JZL195, an irreversible MAGL inhibitor. Each point represents the mean ± S.E.M of values from 3 to 6 separate experiments. * *p* = 0.0126, **** *p* < 0.0001 compared to drug vehicle at the same time after treatment (two-way ANOVA followed by Tukey’s multiple comparison test, Nonlin fit [inhibitor] vs. response [three parameters]).

At a concentration of 1 μM, NGR1 demonstrated a time-dependent, reversible inhibitory effect on the activity of human recombinant MAGL, in contrast to JZL195, which caused an irreversible inhibition of human recombinant MAGL activity (Figure 5). At 2 min of incubation, the magnitude of inhibition was JZL195 (62% inhibition) > NGR1 (41% inhibition), while at 30 min it was JZL195 (64% inhibition) > NGR1 (7.4% inhibition).

**Figure 6:**
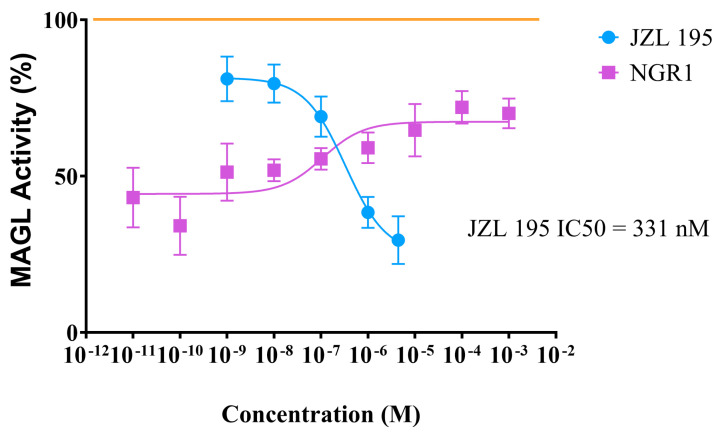
Inhibitory effects of various concentrations of NGR1 and JZL195 on human recombinant MAGL after 2 min of incubation. Each point represents the mean ± S.E.M of values obtained from 3 to 6 separate experiments (Nonlin fit [inhibitor] vs. response [three parameters]).

NGR1 exhibited a concentration-dependent inhibition in MAGL activity at the 2 min in the reverse order, i.e., lost magnitude of inhibition with increased concentration. At the 2 min mark and at lower concentrations of NGR1, MAGL activity was inhibited by approximately 60%; however, as the concentration increased the inhibition of MAGL activity decreased. In contrast, JZL195 inhibited human recombinant MAGL in a concentration-dependent manner with a median inhibitory concentration (IC_50_) of 331 nM (Figure 6).

### 2.4. Effect of NGR1 on Paclitaxel-Induced Monoacylglycerol Lipase Activity in the Paw Skin

**Figure 7:**
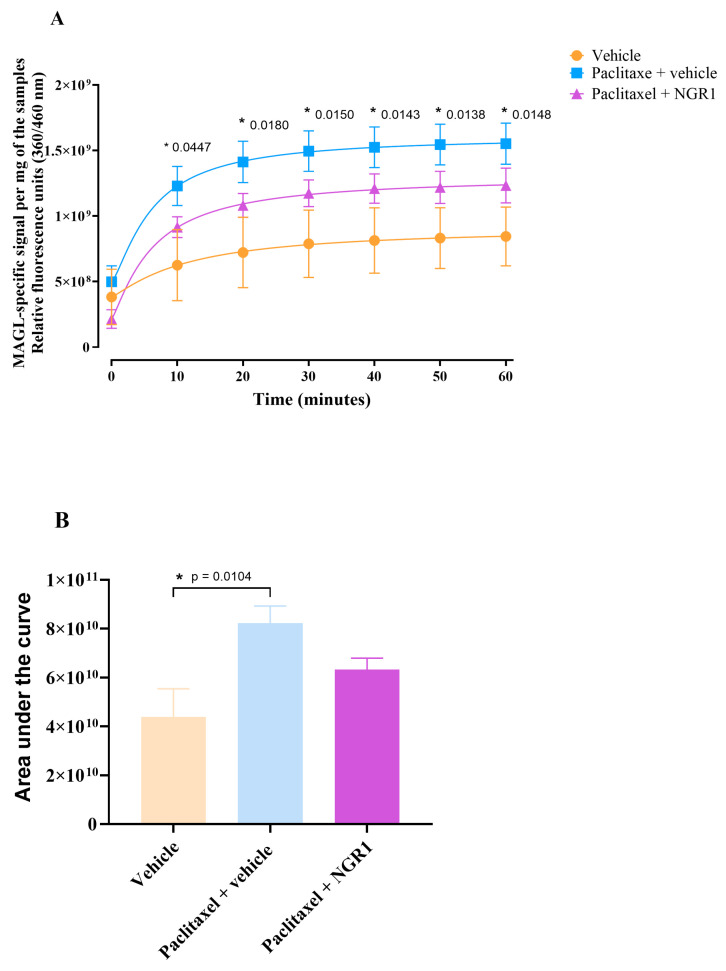
Effect of NGR1 on the paclitaxel-induced increase in MAGL activity in the paw skin of female BALB/c mice. (**A**) Relative fluorescence units (360/460 nm) over time as a product of the MAGL-specific signal per mg of the paw skin samples from vehicle-, paclitaxel-plus-vehicle-, and paclitaxel-plus-NGR1-treated mice at day 7 after the first injection (dpi) of paclitaxel. Each symbol represents the mean ± S.E.M of values obtained from 7 separate experiments from samples pooled from 3 different sets of animals. Each set had 4–7 animals per treatment group. * *p* < 0.05 compared to mice treated with the vehicle only (two-way repeated measures ANOVA followed by Tukey’s multiple comparisons test). (**B**) Area under the curve (AUC) of relative fluorescence of MAGL-specific signal per mg of the paw skin samples from vehicle-, paclitaxel-plus-vehicle-, and paclitaxel-plus-NGR1-treated mice at day 7 dpi of paclitaxel. Each bar represents the mean ± S.E.M of values obtained from 7 separate experiments from samples pooled from 3 different sets of animals. Each set had 4–7 animals per treatment group. * *p* < 0.05 compared to mice treated with the vehicle only (one-way ANOVA followed by Tukey’s multiple comparisons test).

Treatment with paclitaxel resulted in a significant increase in MAGL activity as compared to the vehicle group. The relative fluorescence unit (360/460 nm) of the MAGL-specific signal per 1 mg protein of the paclitaxel group was significantly higher than the vehicle group (Figure 7A). At 10 min, the MAGL-specific signal per 1 mg protein of the paclitaxel group was significantly elevated when compared to vehicle group at the same time point. The MAGL signal in the paclitaxel group continued to rise during the incubation period (up to 60 min). Treatment with NGR1 did not significantly alter MAGL activity as compared to the vehicle-treated and paclitaxel-treated groups. However, the NGR1-treated group showed a trend toward reduction in MAGL activity when compared to the paclitaxel group. The area under the curve (AUC) of MAGL activity was significantly higher in paclitaxel-treated mice (8.3 × 10^10^ ± 6.9 × 10^9^) compared to vehicle-treated mice (4.4 × 10^10^ ± 1.1 × 10^10^); see Figure 7B (mean difference of −3.8 × 10^10^; 95.00% CI of diff. of −6.8 × 10^10^ to −8.7 × 10^9^, *p* = 0.0104). Paclitaxel nearly doubled the activity of MAGL as compared to the vehicle-treated group. Paclitaxel plus NGR1-treated mice (6.3 × 10^10^ ± 4.7 × 10^9^) were not significantly different in the AUC of MAGL activity as compared to paclitaxel plus vehicle-treated mice (8.3 × 10^10^ ± 6.9 × 10^9^) although there was a downward trend (mean difference of 1.9 × 10^10^; 95.00% CI of diff. of −1.1 × 10^10^ to 4.9 × 10^10^; and *p* = 0.2526). The changes in the AUC of MAGL activity in the NGR1-treated group were also not significant when compared to the vehicle-treated group (mean difference of −1.9 × 10^10^; 95.00% CI of diff. of −4.9 × 10^10^ to 1.0 × 10^10^; *p* = 0.2475).

## 3. Discussion

Our current study shows that NGR1 prevents the development of PIMA in female BALB/c mice possibly partially through the inhibition of MAGL activity. NGR1 caused a dose-dependent alleviation of PIMA. In addition, NGR1 produced a reversible, time-dependent, and concentration-dependent inhibition in human recombinant MAGL activity with a paradoxical inhibition pattern and had a trend towards diminishing the paclitaxel-induced increase in MAGL activity in the mouse paw skin.

Paclitaxel has been reported to induce thermal hyperalgesia and cold and mechanical allodynia in mice [15]. In our experiments, female BALB/c mice developed mechanical allodynia following five consecutive days of intraparietal injections of paclitaxel, consistent to previous published studies [9,16].

Pre-treating mice with NGR1 alone for five consecutive days before paclitaxel administration did not have an effect on mice’s response to mechanical stimuli. However, pre-treating mice with NGR1 before and during paclitaxel treatment for a total of 10 days resulted in a dose-dependent improvement in the withdrawal threshold as compared to the paclitaxel-treated group at the same time point. Furthermore, the withdrawal thresholds of NGR1-treated groups were similar to vehicle-treated mice. Thus, NGR1, a triterpenoid secondary plant metabolite, prevents the development of PIMA in female BALB/c mice, similar to what was observed with another triterpenoid, pristimerin [9]. This is in line with the neuroprotectant effects of NGR1 observed in other models in previous studies. In vitro, NGR1 promoted cell growth, improved cellular viability, and reduced apoptosis of neurons following insults with glutamate [17,18], H_2_O_2_ [19], and bupivacaine [20]. In vivo, NGR1 attenuated isoflurane-induced neurological impairment [21] and sevoflurane-induced neurotoxicity [22] by decreasing neuroinflammation and apoptosis, respectively. In addition, NGR1 improved neurological function and locomotion recovery and alleviated spinal cord injury in rats by inhibiting neuronal apoptosis and inflammation [12].

NGR1 may provide an added advantage when combined with paclitaxel because of its anticancer activities. NGR1 has been shown to have anticancer properties in a variety of cancer types including breast cancer [23,24] and non-small lung cancer [25]. Therefore, adding NGR1 to paclitaxel could produce an additive or synergistic on the cancer itself but at the same time protect against neuropathy induced by paclitaxel, a common side effect of paclitaxel that significantly affects the patients’ quality of life.

Using NGR1 up to 10 mg/kg did not cause any sudden change in weight or obvious adverse effect at the end of the experiment. This is in line with a previous study, which reported that higher doses of NGR1 (30 mg/kg) did not cause toxic effects or side effects to normal mice in terms of “body weight change rate, liver weight, serum ALT, serum AST and food intake levels” [26].

Multiple plant-derived MAGL inhibitors have been identified. Both pristimerin, a pentacyclic triterpene, and euphol, a tetracyclic triterpene, were first to be identified to inhibit purified rat recombinant MAGL in vitro [14]. Recently, we reported that pristimerin inhibited human recombinant and mouse paw skin MAGL activity in a concentration-dependent manner [9]. Pristimerin also prevented the development of PIMA and mitigated the concurrent rise in MAGL activity observed in the paw skin [9].

Since NGR1 is a triterpene, similar to pristimerin and euphol, it could have an effect on MAGL activity comparable to pristimerin and euphol. Indeed, our molecular docking results have shown that NGR1 has a good affinity, though lower than pristimerin, to MAGL suggesting that NGR1 could bind to and inhibit MAGL activity like pristimerin. These in-silico predictions were subsequently supported by experimental validation using a human MAGL screening assay. In vitro experiments with recombinant human MAGL revealed that NGR1 produced a reversible inhibition of the enzyme. Interestingly, this inhibition was concentration-dependent though in a reverse pattern, i.e., the lower concentrations of NGR1 had higher MAGL inhibition than the higher concentrations. Our findings are consistent with the concept of non-linear, biphasic dose–response relationships, often represented as J- or U-shaped (hormetic) curves. In this model, low concentrations exert inhibitory effects, whereas higher concentrations lead to stimulation. Such non-monotonicity likely reflects underlying mechanisms such as enzyme modulation [27,28]. This modulation may reflect an initial allosteric or stabilizing interaction between NGR1 and MAGL at low doses, whereas at higher concentrations, conformational changes, or partial competition with the substrate could reduce inhibitory efficiency. More enzyme kinetics experiments are needed to fully elucidate the nature of enzyme inhibition by NGR1.

In addition to NGR1’s ability to inhibit human MAGL, NGR1 could mitigate paclitaxel-induced increases in MAGL activity. Consistent with previous studies [9], paclitaxel increased MAGL activity in the paw skin of mice suggesting that the development of PIMA may be due in part to the elevation in MAGL activity in the periphery. NGR1 treatment did not significantly change MAGL activity as compared to paclitaxel or vehicle groups although there was a trend toward reducing the paclitaxel-induced rise in MAGL activity in the paw skin of female BALB/c mice. This alone cannot explain the protection effect of NGR1 in PIMA; thus, other mechanisms are most likely involved in the NGR1’s ability to prevent PIMA.

Other underlying mechanisms may also be responsible for the NGR1 protection effect in PIMA. In addition to probable alteration in MAGL activity, NGR1 could also prevent the development of PIMA possibly through its antioxidant, antiapoptotic, and anti-inflammatory activities [29,30]. The pathogenesis of CINP involves multiple mechanisms other than dysregulation in ECS. It may involve activation of oxidative stress, apoptosis, and inflammatory pathways [3]. The heightened status of these pathways has been reported in CINP. Generation of reactive oxygen radical species and depletion of antioxidant defenses such as nuclear erythroid factor-2 related factor (NRF2), superoxide dismutase 2 (SOD2), and heme oxygenase 1 (HO1), in addition to the release of proapoptotic mediators and inflammatory cytokines, contribute largely to peripheral nerve damage in CINP [3]. NGR1’s previously reported neuroprotective effects were produced by inhibiting oxidative stress through activation and upregulation of NRF2 and subsequent downstream effectors such as HO1, Nicotinamide adenine dinucleotide phosphate (NAD(P)H), quinone oxidoreductase-1, SOD, and glutathione peroxidase (GSH-PX) [12,19,22]. In addition, NGR1 diminished neuronal apoptosis either by reducing pro-apoptotic markers such as caspase-3, caspase-9, and Bax or by stimulating anti-apoptotic markers such as Bcl-2 [12,19]. Moreover, NGR1 protected against neurological impairment by preventing inflammatory markers such as interleukin-6 (IL-6), IL-1β, and tumor necrosis factor-alpha (TNF-α) [21]. Therefore, it is plausible that NGR1 may have also prevented the development of PIMA in the current study through similar mechanisms. Whether NGR1 could affect the expression, activity, and levels of the abovementioned mediators in PIMA merit further investigation.

Major limitations of this study were the use of female mice only. Including both sexes could have allowed for a comparative analysis to assess whether there are sex-specific variances in the underlying mechanisms of NGR1’s prevention of PIMA. However, due to constraints in the availability of animals, this aspect could not be fully addressed in the present study. In addition, experiments were not blinded, and this may introduce bias. Moreover, while the study focused on the impact of NGR1 on MAGL activity in PIMA, other underlying mechanisms may also contribute to NGR1’s protective effects. Other possible mechanisms including anti-inflammatory, antioxidant, and anti-apoptotic pathways could be involved, and further investigations are recommended to further explore these pathways and their involvement in NGR1’s ability to prevent the development of PIMA.

**Figure 8:**
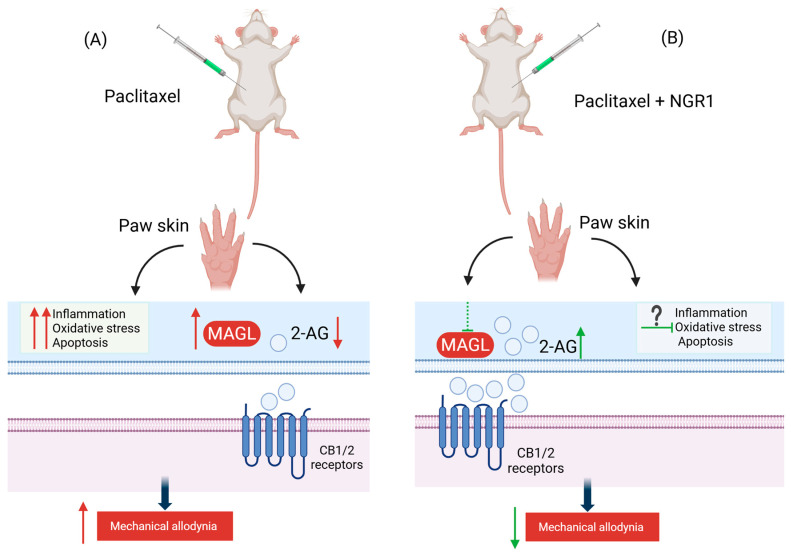
A schematic representation of the antiallodynic effect of NGR1 in a mouse model of paclitaxel-induced mechanical allodynia. NGR1 prevents the development of PIMA. Multiple pathways could potentially be involved in NGR1-induced alleviation in mechanical allodynia in female mice. (**A**) In a female mouse model of PIMA, the intraperitoneal injection of paclitaxel increases MAGL activity in paw skin of mice. The increase activity of MAGL would enhance the degradation of 2-AG and thus lead to the reduction in the levels of 2-AG. Consequently, low levels of 2-AG could reduce the activation of the CB1 and CB2 receptors resulting in increased sensitivity to mechanical allodynia. Paclitaxel also activates pathways involved in oxidative stress, apoptosis, and inflammation (**B**) Prophylaxis treatment with NGR1 before the development of PIMA could potentially reduce the paclitaxel-associated increases in MAGL activity and thus decrease the degradation and increase the availability of 2-AG to bind to CB1 and CB2 receptors. Stimulation of these receptors could reduce the mechanical allodynia induced by paclitaxel and subsequently elicit the antiallodynic and analgesic effect of NGR1. Other pathways such as anti-inflammatory, anti-apoptotic, and antioxidant could also possibly be activated and involved in NGR1 antiallodynic and analgesic actions. The exact contribution needs further experimental confirmation. 2-AG: 2-arachidonoyl glycerol; MAGL: monoacylglycerol lipase; NGR1: Notoginsenoside R1; PIMA: paclitaxel-induced mechanical allodynia, (Created by biorender.com).

In conclusion, this proof-of-concept preclinical study demonstrates, for the first time, the potential of NGR1 to prevent CINP and possibly modulate MAGL activity, although this may not be its sole mechanism of action. The proposed mechanism by which NGR1 exerts its antiallodynic action is depicted in the schematic diagram in Figure 8. NGR1 inhibited human recombinant MAGL activity in a time-dependent and reversible manner, though the concentration-dependent effect was in reverse order, i.e., less inhibition with increased concentration suggests a hormetic-like effect. It also effectively prevented PIMA and caused a non-significant downward trend in reducing the associated increase in MAGL activity in the paw skin of mice. These findings suggest that NGR1 could be a promising candidate for mitigating paclitaxel-induced neuropathic pain. Future research should aim to elucidate additional molecular mechanisms underlying NGR1’s effects on PIMA, incorporate male subjects alongside females, and employ diverse models of CINP beyond paclitaxel-induced neuropathic pain, with particular emphasis on assessing functional recovery and the long-term efficacy of NGR1.

## 4. Materials and Methods

### 4.1. NGR1 Preparation

Notoginsenoside R1 (NGR1) was purchased from Sigma (N3915-10 mg, Darmstadt, Germany, purity ≥ 98%) and was dissolved in sterile water to prepare a stock of 1 mg/mL. The mixture was then stored at −20 °C for further experiments.

### 4.2. Animals

This study utilized female BALB/c mice aged 8–12 weeks (*n* = 91), obtained from the Animal Resources Centre (ARC) at the Health Sciences Centre (HSC), Kuwait University. The decision to exclusively employ female mice aligns with the recommendation from the Sex, Gender, and Pain Special Interest Group (SIG) of the International Association for the Study of Pain (IASP). This group suggests that pain researchers “should test their hypotheses in both sexes or, if limited by practical constraints, focus solely on females” [31]. The selection of the BALB/c strain was based on its availability and our previous research experience with the same strain [8,9,16,32]. Animal handling adhered to the guidelines of Directive 2010/63/EU of the European Parliament and of the Council concerning the protection of animals used for scientific purposes. Approval for all animal experiments was granted by the Ethical Committee for the Use of Laboratory Animals in Teaching and in Research, HSC, Kuwait University (Ref: PG-24-10. Date: 09/01/2024). The mice were housed in temperature-controlled rooms (22 ± 1 °C) with ad libitum access to food and water. To mitigate the influence of circadian variations in pharmacological effects, all experiments were conducted between 800 and 1600 h.

### 4.3. Induction of PIMA in Mice

A paclitaxel (Tocris, Bristol, UK) stock solution (6 mg/mL) was prepared by dissolving paclitaxel in a mixture of 50% Cremophor EL and 50% absolute ethanol. The solution was stored at −20 °C for up to 14 days. On the day of the experiment, the stock solution was diluted to a final concentration of 0.2 mg/mL in normal saline (0.9% NaCl) immediately before injection. The vehicle, consisting of the same Cremophor EL and ethanol mixture, was similarly diluted with normal saline in the same proportion as the paclitaxel solution at the time of injection. Mice received intraperitoneal (i.p.) injections of paclitaxel (2 mg/kg) or its vehicle at a volume of 10 μL/g body weight, administered once daily for five consecutive days [16].

### 4.4. Drug Administration Protocol

**Figure 9:**
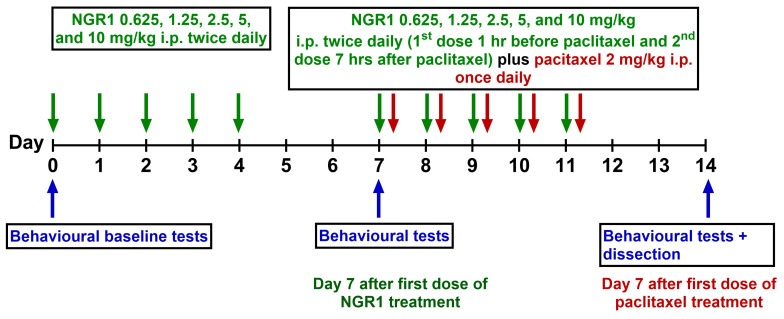
Schematic representation of paclitaxel and drug administration protocols. The green arrows indicate the days when NGR1 was administered. NGR1 was administered to the mice by intraperitoneal injection 5 days before and 5 days during paclitaxel injection. The red arrows indicate the days when paclitaxel was administered. The blue arrows indicate the days when behavioral tests were performed.

NGR1 was administered i.p. at a dosage range from 0.625 to 10 mg/kg twice daily for five consecutive days, beginning seven days prior to the initiation of paclitaxel administration. This was followed by an additional administration of NGR1 at the same dose ranges (0.625–10 mg/kg) for another five days one hour before the injection of paclitaxel. The next injection of NGR1 was 7 h after the paclitaxel injection. Control animals received the vehicle for paclitaxel, comprising ethanol and Cremophor EL, following the same injection pattern as paclitaxel. The control and paclitaxel animals were also administered the vehicle for NGR1, distilled water, following the same administration pattern as NGR1 (Figure 9). All behavioral studies were not blinded. The initial distribution and randomization of animals into different groups was based on their baseline behavioral values at day −7.

### 4.5. Assessment of Mechanical Allodynia

Mechanical allodynia was assessed as previously prescribed [9,16] utilizing the dynamic plantar aesthesiometer (Ugo Basile, Gemonio, Italy). In brief, mice were allowed to acclimatize for 30–60 min inside plastic enclosures positioned on a perforated platform. A microprocessor, programmed to automatically raise a metal filament applying a linearly increasing force (0.25 g/s with a cut-off time of 20 s) on the hind paw, was initiated when the mice were settled. The recording automatically stopped when the animal withdrew the paw or was stopped when the cut-off force of 5 g was reached. Withdrawal thresholds in response to the mechanical stimuli were automatically recorded in grams. The hind paws underwent testing at least three times with a 2 min rest when measuring the same paw.

### 4.6. Tissue Dissection

Following behavioral measurements at day 7 after the first paclitaxel injection, mice were anesthetized with halothane and sacrificed by decapitation. The skin from both hind paws was carefully dissected, immediately frozen in liquid nitrogen, and stored at −80 °C for measurement of mouse MAGL activity experiments.

### 4.7. Molecular Docking

The PubChem database (https://pubchem.ncbi.nlm.nih.gov/) was used to search for and download the 3-dimensional (3D) structures of notoginsenoside R1 (NGR1, PubChem CID 441934) and pristimerin (Pub-Chem CID 159516). The structures were downloaded in .sdf 3D conformer format.

The Protein Data Bank (PDB, https://www.rcsb.org/) was used to search for and download the 3D structure of human MAGL (PDB id 5ZUN). The structure was downloaded in .pdb format. The molecular docking of the ligands to the protein was performed using the structure-based blind docking option on an online docking server, CB-Dock2 from the Yang Cao lab (https://cadd.labshare.cn/cb-dock2/index.php, accessed on 5 February 2025), as previously described [33].

### 4.8. Assessment of Inhibitory Potential of NGR1 on Human Recombinant Monoacylglycerol Lipase Activity

The effect of NGR on the activity of human recombinant MAGL was assessed in vitro using the MAGL assay inhibitor screening colorimetry-based kit (Cayman Chemical, Ann Arbor, MI, USA) as previously described [9], following the manufacturer instructions. The MAGL enzyme hydrolyses the substrate (4-nitrophenyl acetate), producing 4-nitrophenol, whose absorbance was measured at 405 nm using a microplate reader. For the assay, JZL195, an inhibitor of MAGL, was used and served as a reference standard. NGR1 and JZL195 were prepared in a solution comprising 50% dimethylsulfoxide and 50% assay buffer. A mixture of assay buffer (150 μL), diluted human recombinant MAGL (10 μL), and either a solvent or inhibitor (10 μL) at different concentrations was incubated for 15 min at 25 °C in a 96-well microplate. The reaction was initiated with the addition of 10 μL of MAGL substrate to all wells. The plate was shaken for 10 s before absorbance at 405 nm was measured at different time points (2, 5, 10, and 30 min) using a microplate reader (SpectraMax^®^ iD3, Molecular Devices, San Jose, CA, USA). The %Activity was calculated as Inhibitor activity/Initial activity ×100, following the manufacturer’s protocol. Initial activity represents the absorbance in wells where the reaction occurred without an inhibitor.

### 4.9. Assessment of Inhibitory Potential of NGR1 on Monoacylglycerol Lipase Activity in Mouse Paw Skin Tissue

The effect of NGR1 on MAGL activity in mouse tissue was assessed in vitro using the MAGL activity fluorometric based-assay kit (Abcam, Waltham, MA, USA) as previously described [9], following the manufacturer instructions. The assay involves MAGL cleaving a fluorescent substrate to produce arachidonic acid and a fluorescent metabolite, with increased fluorescence measured at Ex/Em 360/460 nm. To differentiate MAGL activity from other sources of fluorescence, a proprietary MAGL inhibitor was employed. The dissected paw skin was homogenized using a MAGL assay buffer (provided in the kit), and the homogenate was centrifuged at 10,000 PRM for 15 min. The supernatant was collected into a new 1.5 mL Eppendorf tube and stored at −80 °C until used for MAGL activity assays. The protein concentrations were measured using the Pierce BCA Protein Assay Kit (Invitrogen, Waltham, MA, USA) according to the manufacturer’s instructions. The MAGL activity was quantified using 0.05 mg of protein per reaction. A mixture of assay buffer (60 μL), mouse paw skin supernatant (20 μL), and a solvent or inhibitor (10 μL) was incubated for 30 min at 37 °C in a 96-well black microplate. The reaction was initiated by adding 10 μL of MAGL substrate to all wells, and the plate was incubated at 37 °C in a microplate reader (SpectraMax^®^ iD3, Molecular Devices) set at low, 1 OD (optical density) and an integration at 140 ms. The fluorescence measurement was conducted using Ex/Em 360/460 in kinetic mode for 60 min (reading every 10 min); the plate was shaken for 5 s before each measurement to ensure mixing.

### 4.10. Statistical Analysis

The data was normally distributed; thus, data is represented as mean ± S.E.M. Differences between treatments groups were assessed using one-way or two-way analysis of variance (ANOVA) and Tukey’s or Dunn’s multiple comparison test using GraphPad Prism software (version 10.0), and the differences were considered significant at *p* < 0.05.

## Figures and Tables

**Table 1:** Molecular docking results of NGR1 and pristimerin on MAGL enzyme obtained from CB-Dock2. The shared amino acid contact residues the ligands bound to are highlighted in red.

Triterpene	Vina Score (kcal/mol)	Cavity Volume (Å3)	Center (x, y, z)	Docking Size (x, y, z)	Contact Residues
NGR1	−7.8	1904	−10, 17, −6	28, 28, 28	GLY50 ALA51 GLY52 GLU53 MET88 HIS121 SER122 MET123 GLY124 LEU148 ALA151 ASN152 PRO153 GLU154 SER155 ALA156 THR158 PHE159 LEU162 SER175 SER176 GLY177 PRO178 ILE179 ASP180 SER181 VAL183 LEU184 TYR194 ALA203 GLY204 LEU205 LYS206 PHE209 GLY210 ILE211 LEU213 LEU214 VAL217 ALA238 ARG240 LEU241 CYS242 ASP243 HIS269 VAL270
Pristimerin	−10.3	1904	−10, 17, −6	23, 23, 23	GLY50 ALA51 MET88 SER122 MET123 LEU148 ALA151 SER155 ALA156 PHE159 SER176 GLY177 PRO178 ILE179 ASP180 VAL183 LEU184 LEU205 GLY210 LEU213 LEU214 VAL217 ARG240 LEU241 HIS269

## Data Availability

The original contributions presented in this study are included in the article. Further inquiries can be directed to the corresponding author.

## Abbreviation

2-AG	2-arachidonoyl Glycerol
3D	3-dimensional
AEA	Anandamide or N-arachidonoylethanolamide
ANOVA	Analysis of Variance
ARC	Animal Resources Centre
CB1	Cannabinoid Receptor type 1
CB2	Cannabinoid Receptor type 2
CIPN	Chemotherapy-induced Peripheral Neuropathy
ECS	Endocannabinoid System
FDA	Food and Drug Administration
GSH-PX	Glutathione Peroxidase
HO1	Heme Oxygenase 1
HSC	Health Sciences Center
i.p.	Intraperitoneal
IASP	International Association for the Study of Pain
IC50	Inhibitory Concentration
IL	Interleukin
MAGL	Monoacylglycerol Lipase
NGR1	Notoginsenoside R1
NRF2	Nuclear Factor Erythroid 2-related Factor 2
OD	Optical Density
PIMA	Paclitaxel-induced Mechanical Allodynia
PINP	Paclitaxel-induced Neuropathic Pain
SOD2	Superoxide Dismutase 2
TNF-α	Tumor Necrosis Factor-alpha
